# Stress-correlated stereom organization in the echinoid tubercle reveals hierarchical mechanical integration

**DOI:** 10.1098/rsif.2026.0592

**Published:** 2026-10-14

**Authors:** Timon Gehrmann, Przemyslaw Gorzelak, Susanna Labisch, Jan-Henning Dirks

**Affiliations:** ^1^ Department of Biomimetics, Hochschule Bremen – City University of Applied Sciences, Bremen, Germany; ^2^ Biomimetics-Innovation-Centre, Hochschule Bremen – City University of Applied Sciences, Bremen, Germany; ^3^ Institute of Paleobiology, Polish Academy of Sciences, Warsaw, Poland

**Keywords:** functional morphology, stress–structure correlation, finite-element analysis, X-ray microscopy, echinoids

## Abstract

Echinoid tests consist of a highly porous skeletal microstructure (stereom) optimized in terms of functional performance. One of the most mechanically demanding components of the test is the tubercle, which forms the mechanical interface between the test plates and spines and is subjected to complex loads generated by motion of the spine. However, the mechanical behaviour of tubercles under realistic loading conditions remains poorly explored. Here, we investigate the tubercle of *Prionocidaris baculosa* using high-resolution X-ray microscopy and finite-element analysis to simulate physiologically relevant loading, identify the main stress directions, and assess mechanical efficiency through topology optimization. By correlating experimental and numerical results, we quantify the relationship between stress distributions and stereom organization, providing new insights into the mechanical complexity of echinoid skeletons. We found a strong correlation between experimentally determined density and different stress parameters (von Mises stress, principal stress trajectories and mean normal stress), supporting the hypothesis of a functionally graded and mechanically optimized stereom architecture. These findings highlight the echinoid skeleton as a naturally optimized, load-adaptive structure with potential relevance for bioinspired design. In particular, stereom-inspired architectures could inform additive manufacturing approaches, enabling lightweight and material-efficient components with tailored mechanical performance derived from a single material.

## 1. Introduction

Echinoderms possess one of the most distinctive skeletal systems in the animal kingdom—an endoskeleton made of single-crystalline calcite plates intricately organized into a porous, three-dimensional framework, called stereom [1,2]. This skeletal microstructure constitutes a key synapomorphy of the phylum and is already present in the earliest echinoderm fossils from the Cambrian, indicating that this biomineralized microarchitecture originated very early in echinoderm evolution and has been conserved for over 500 million years [2]. These natural skeletal systems thus offer unique opportunities to study how biomineralized architectures can combine mechanical competence, developmental plasticity and functional versatility within a single material system. Unlike most invertebrate exoskeletons, echinoderm ossicles grow as living parts of the organism, making them exemplary models for understanding how structural complexity emerges from biological and physicochemical principles.

Among echinoderms, sea urchins (Echinoidea) have become central models for exploring the relationship between mineral microstructure and function [3–8]. Their calcitic ossicles may exhibit a highly organized porous microarchitecture [9–11], which represents a continuous network of trabeculae and cavities varying in size, orientation and connectivity. The stereom is not merely a structural scaffold, but an integral component of the living tissue system, participating in both mechanical support and physiological exchange. Studying its organization provides insight into the coupling between form, function and material properties under evolutionary pressure in one of the most ancient deuterostome skeletons.

The echinoderm stereom comprises a range of morphologically distinct types, each hypothesized to serve specialized functions within the ossicle [1,12]. Observations of their distribution patterns within ossicles suggest that these structures have undergone functional differentiation across regions of varying mechanical stress. For example, galleried stereom typically occurs at ligament anchorage points, fine labyrinthic stereom at muscle attachment sites, microperforate or imperforate stereom in regions under high compressive stress, and triply periodic minimal surface (TPMS)-like stereom in regions requiring multi-directional load accommodation [1,4,13–16]. These spatial patterns imply that the architecture of stereom may reflect both evolutionary adaptation and local mechanical demands.

Recent studies have provided evidence supporting this interpretation. For example, Perricone *et al.* demonstrated that the galleried stereom of the common sea urchin *Paracentrotus lividus* exhibits a Voronoi-like organization that probably enhances both mechanical stability and growth efficiency [17]. Similarly, Raman *et al.* [18] reported that in *Asterias rubens* the ultrastructure of ossicles is correlated with mechanical load. However, unlike vertebrate bone, in which the trabeculae align primarily along the principal stress trajectories [19], echinoderm skeletons display a striking diversity of distinct, interrelated porosity patterns that probably serve multiple overlapping functions beyond mechanical optimization [1,16]. Despite this complexity, quantitative analyses linking local stress distributions to specific stereom morphologies remain limited. Notably, Chen *et al.* performed the first detailed quantitative analysis of the three-dimensional architecture of *Heterocentrotus mammillatus* spines, revealing a functionally graded structure with increasing density and mechanical isotropy from centre to periphery—an adaptation probably related to bending resistance [20,21].

Among the most mechanically demanding components of the echinoid test is the tubercle, which forms the mechanical interface between test plates and spines, transmitting complex compressive, tensile and bending loads generated during spine movement, with attachment mediated by catch connective tissue [22–25]. Despite its functional importance, the mechanical behaviour of tubercles under realistic loading conditions remains poorly explored. One of the few detailed studies to date by Ji *et al.* demonstrated that the echinoid tubercle exhibits a core–shell architecture, consisting of a dense outer cortex surrounding an inner, highly ordered stereom with uniformly distributed, rounded pores [26]. The latter authors further proposed that this hierarchical organization underpins the mechanical performance of the tubercle, as the stereom displays a periodic macroscale arrangement and axially aligned, bottleneck-shaped trabeculae whose characteristic dimensions reduce stress concentrations and enhance fracture resistance and energy dissipation at relatively high porosity. To fill this gap, the present study investigates the stereom organization and mechanical performance of the tubercle in the cidaroid sea urchin *Prionocidaris baculosa*. This structure, which functions as a ball-and-socket joint connecting the spine to the test, represents an exemplary case of a biological system that combines local specialization with structural integration.

The spines of sea urchins contribute to locomotion, defence, and sensory perception [12,13,24]. Consequently, the tubercle must withstand diverse mechanical stresses while maintaining mobility and strength. Mechanically, the tubercle–spine system is supported by muscles and ligaments that allow controlled movement and provide stability to the joint. The muscle ring acts as an actuator, allowing the spine to pivot in multiple directions, while the internal and external collagenous ligaments guide and limit movement, preventing joint displacement under load (figure 1A,B). Internal ligaments are typically found in larger or more heavily loaded tubercles, where they provide additional reinforcement. The tubercle itself comprises several subregions: the mamelon (the spine attachment site), the platform (subdivided into crenulate and smooth zones providing torsional resistance or greater mobility, respectively), the boss (the external ligament attachment site) and the areole (the muscle attachment site) [1]. The maximum angular deflection of the spine typically ranges from $25^{\circ }$ to $30^{\circ }$, measured from the vertical axis.

**Figure 1.**
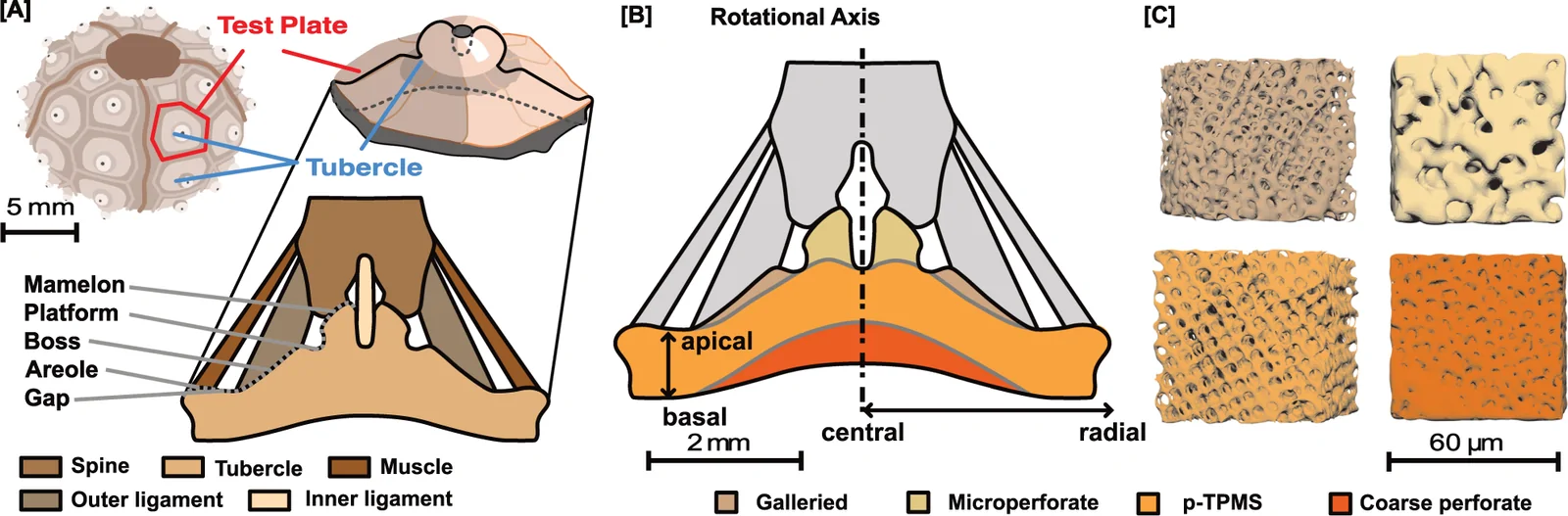
Structural organization of the echinoid tubercle system. (A) Overall skeletal structure showing the arrangement of ossicles and test plates. Furthermore the tubercle–spine–muscle–ligament system is to be seen. (B) Distribution of different stereom types within a longitudinal cross-section of a *Prionocidaris baculosa* tubercle. (C) Stereom morphologies observed in the tubercle of *Prionocidaris baculosa*.

Like other ossicles, the tubercle exhibits a complex internal ultrastructure composed of multiple stereom types arranged according to functional zones. In *Prionocidaris*, for example, imperforate/microperforate, rectilinear, galleried and irregular perforate stereoms coexist within a single plate, forming a finely graded microarchitecture (figure 1C). The rectilinear stereom described by [1] has since been demonstrated to display TPMS geometries, and subsequent studies have identified TPMS variants—such as diamond-shaped and primitive forms—in echinoids, crinoids and starfish [11,27–31]. Primitive TPMS stereom, which is later called p-TPMS, appears to be the most common, representing an efficient compromise between low density and strength.

Looking at the complex internal structure of the tubercle of *Prionocidaris baculosa*, we hypothesize that the different stereom types might be functionally specialized and mechanically optimized to accommodate different loading scenarios. To test this, we combined high-resolution X-ray microscopy (XRM) to characterize the internal microarchitecture with finite-element analysis (FEA) to simulate mechanical loading, perform topology optimization and determine principal stress directions [32,33]. These methods were used to evaluate whether the natural stereom topology and orientation reflect mechanically efficient configurations. By correlating experimental and simulated data, we established quantitative links between the distribution of stresses and the organization of stereom types, thereby elucidating the principles underlying the remarkable mechanical complexity of echinoid skeletons.

## 2. Material and methods

### 2.1. Specimens

Multiple specimens of the cidaroid sea urchin *Prionocidaris baculosa* were obtained from the Institute of Paleobiology (Polish Academy of Sciences, Warsaw, Poland), originating from the coastal region of Cebu, Philippines (10${}^{\circ }$ 14${}^{\prime }$ N, 123${}^{\circ }$ 53${}^{\prime }$ E). All specimens were first screened by micro-CT to assess structural integrity and the degree of radial symmetry of the tubercle. The specimen exhibiting the most complete and geometrically symmetrical tubercle was selected for subsequent high-resolution XRM imaging and FEA. A single tubercle–spine assembly was taken to full XRM resolution; combined scan duration for the tubercle and spine exceeded 24 h, precluding routine replication at this resolution within the scope of the present study.

Cidaroid stereom microarchitecture is well documented to be conserved and stereotypic across tubercles within an individual and, to a considerable extent, across related cidaroid taxa [1,11,16], making the analysed assembly representative of a broader structural pattern. The primary aim of this study is to establish a mechanistic framework linking stereom organization to local stress distributions within the tubercle–spine joint, rather than to quantify inter-individual or interspecific variability; the use of a single well-characterized specimen is therefore appropriate for this purpose. The present study establishes the mechanistic baseline upon which future multi-specimen and multi-species comparisons can be built, and we have stated this explicitly as a limitation in the discussion.

Specimens containing soft tissues were dried at 50${}^{\circ }\text{C}$ for 48 h prior to imaging. For XRM scanning, the selected test plate with attached spine was carefully dissected and mounted with the spine vertically oriented in the sample holder.

The plate selected for analysis was an interambulacral primary plate (from the ambitus; ambital test diameter: approx. 20 mm; spine length: approx. 20 mm). Interambulacral tubercles in cidaroids may exhibit reduced crenulation development on one sector of the platform rim, permitting greater angular freedom of the spine relative to more uniformly crenulated tubercles.

### 2.2. X-ray microscopy

High-resolution micro-CT was performed using a Zeiss Xradia Versa 520 (Zeiss, Oberkochen, Germany, 80 kV, 87 mA, 12 s exposure time, 4× binning 1.12 (μm) voxel size), based on protocols from Schwertmann *et al.* [34]. Volumetric reconstruction was performed in Zeiss Scout-and-Scan v13.

### 2.3. Image processing and density quantification

CT volumes were processed in ImageJ 1.54 g and visualized in CTVox. Central longitudinal sections through the tubercle were used to define three reproducible radial paths (P1 to P3) for quantitative analysis; see figure 2A1. Images were binarized by adaptive thresholding, and brightness values (0–255) were converted to relative density, where 100% corresponds to zero porosity. Density profiles were averaged across five adjacent slices (total depth = 20 voxels) and smoothed with a mean moving-window $20\times 20\times 20\;\mu {\text{m}}^{3}$ (figure 2A2). For these density profiles sixth-order polynomial regressions ($R^{2}=0.88\pm 0.02$) were computed in MATLAB 2022a.

**Figure 2.**
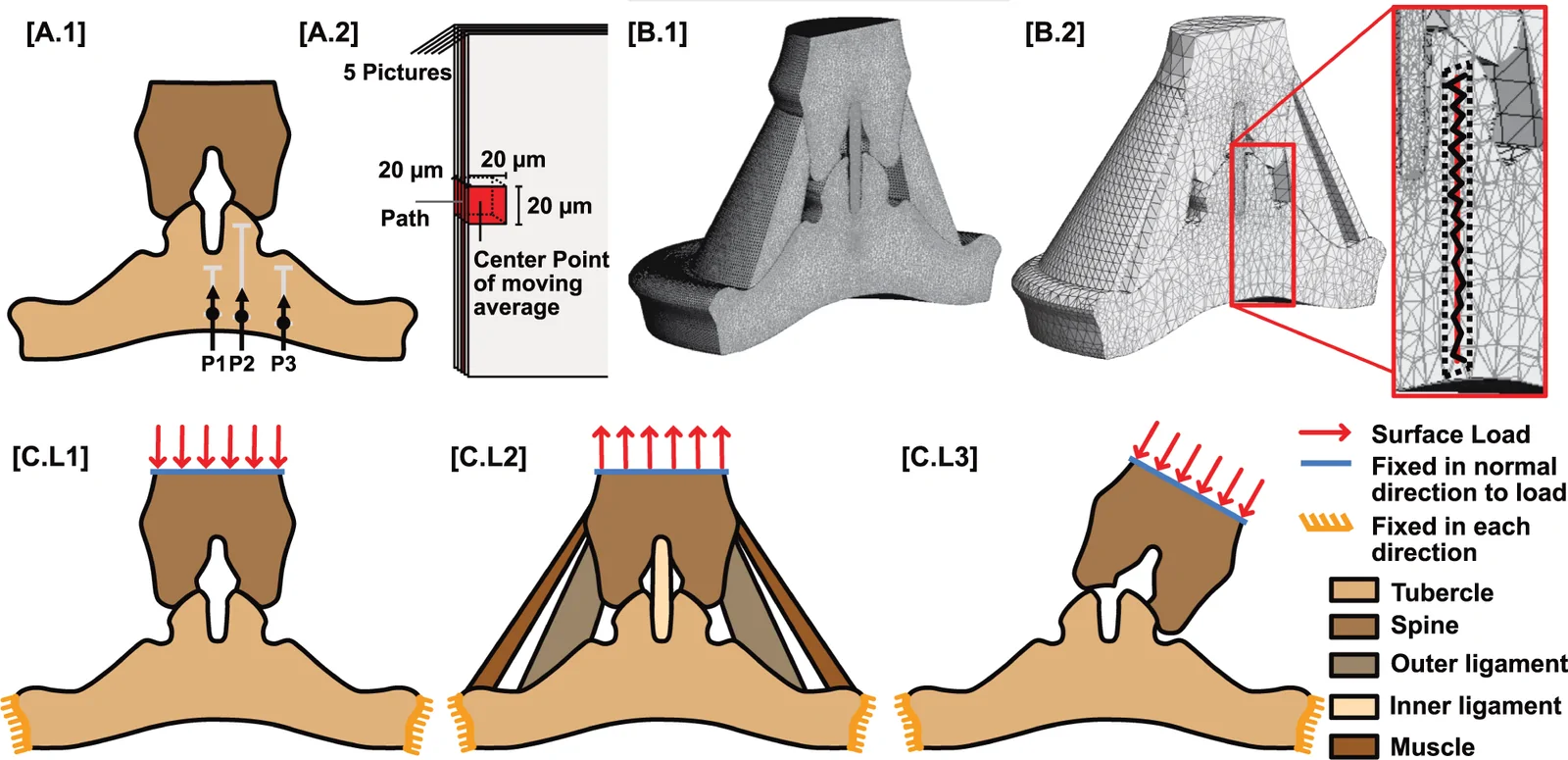
Evaluation of experimentally acquired data and setup for the FEA. (A1) Visualization of the paths through the tubercle evaluated in this study for quantitative correlation. These paths were used consistently for both experimentally measured and numerically derived data to ensure comparability between the two datasets. (A2) For quantifying the XRM data along a path five consecutive images were analysed, corresponding to an approximate depth of 20 μm and a volume of 20 μm × 20 μm × 20 μm. (B1) Finite-element mesh used for the FEA and subsequent structural optimization (SKO). (B2) Mesh used for the CAIO calculation. Owing to the three-dimensional curvature of the mesh, a perfectly straight path was not feasible. Deviations of the nodes from the central path were neglected, as the finer mesh shown in (B1) was used and the deviations were around 10 μm. (C) Overview of the different load cases and contact bodies applied in the numerical simulations. C.L1 shows compressive loading of the spine in the undeformed configuration, C.L2 shows tensile loading of the spine in the undeformed configuration and C.L3 shows compressive loading of the spine in the maximally deflected configuration at $29^{\circ }$ relative to the rotation axis. In C.L1 and C.L3, only the tubercle and spine were modelled, as ligaments and muscles transmit force exclusively in tension. In C.L2, all five contact bodies were included: tubercle, spine, inner ligament (termed ‘central ligament’ by some studies [12]), outer ligament and muscle ring.

### 2.4. Computer-aided design modelling

Three-dimensional models of the tubercle and spine were generated from XRM sections using Autodesk Inventor 2024.2, assuming approximate radial symmetry according to Smith *et al.* [12]. The spine socket was geometrically aligned with the spherical head of the tubercle.

The following load cases are defined under the assumption that muscles and ligaments fulfil their physiological roles of guiding and constraining spine motion within the joint.

Three mechanically relevant configurations were prepared: the upright ($0^{\circ }$) configuration of the tubercle and spine (L1), the upright configuration including muscle and ligament attachments (L2), and a maximally deflected configuration at $29^{\circ }$ relative to the rotational axis (L3). The $29^{\circ }$ deflection angle was determined geometrically from the three-dimensional computer-aided design (CAD) reconstruction of the specific tubercle under investigation: the platform rim geometry, and in particular the arrangement of crenulations, physically constrains the maximum angular displacement of the spine, and this geometric limit was measured directly from the reconstructed model. The value is consistent with the morphological range of 25–$30^{\circ }$ reported by Smith *et al.* [12]. Using the deflection angle specific to this tubercle was a deliberate methodological choice: applying a different angle could not guarantee correspondence between the simulated stress field and the actual internal stereom architecture of the assembly analysed.

### 2.5. Finite-element analysis

FEA was performed using MSC Marc/Mentat 2023.1 (Hexagon AB, Stockholm, Sweden). Models were meshed with linear isoparametric three-dimensional tetrahedral elements. Two material formulations were employed: a linear elastic isotropic model for simulations used in the subsequent SKO topology optimization, and a linear elastic orthotropic model for simulations used in the subsequent CAIO orientational optimization (table 1). The mesh for isotropic (1) and orthotropic (2) simulations can be seen in figure 2B.

**Table 1.** Material parameters used in simulations. Here, E denotes Young's modulus, ν Poisson's ratio, and G shear modulus. The isotropic Young's modulus and Poisson's ratio for biological calcite were taken from [4] and [35] respectively. For the orthotropic model, the Young's modulus in the primary load-bearing direction ($E_{1}$) was set equal to the isotropic value; the transverse moduli $E_{2,3}$ were intentionally reduced by approximately one order of magnitude relative to $E_{1}$. These transverse values do not represent the physical anisotropy of the stereom, for which directional stiffness data are not available, but enforce a numerically robust directional stiffness contrast within the CAIO framework. All remaining parameters ($G_{12}$, $G_{23}$, $G_{31}$, $\nu_{12}$, $\nu_{23}$, $\nu_{31}$) were assigned to satisfy the equilibrium conditions for a positive-definite orthotropic stiffness tensor, as required for numerical convergence in MSC Marc/Mentat. The orthotropic material definition serves solely to provide a computational framework for identifying principal stress directions via CAIO; the results are insensitive to the exact values of the transverse moduli provided that a sufficient stiffness contrast between primary and transverse directions is maintained.

material model	E (MPa)	ν	G (MPa)
isotropic	73500	0.40	—
orthotropic	$E_{1}=73\;500$, $E_{2,3}=6000$	$\nu_{12}=\nu_{31}=0.20$, $\nu_{23}=0.35$	$G_{12}=G_{31}=9000$, $G_{23}=8000$

The orthotropic material parameters require clarification. The Young's modulus in the primary load-bearing direction ($E_{1}=73\;500$ MPa) was set equal to the isotropic value from Perricone *et al.* [4]. The transverse moduli ($E_{2}=E_{3}=6000$ MPa) were intentionally reduced by approximately one order of magnitude relative to $E_{1}$; these values do not represent the physical anisotropy of the stereom, for which directional stiffness data are not available, but enforce a numerically robust directional stiffness contrast within the CAIO framework. The remaining elastic constants were selected to satisfy the equilibrium conditions for a positive-definite orthotropic stiffness tensor, which are required for numerical convergence in MSC Marc/Mentat. The purpose of the orthotropic material definition is solely to provide a computational framework for identifying principal stress directions; the CAIO results are insensitive to the exact values of the transverse moduli provided that a sufficient stiffness contrast between the primary and transverse directions is maintained, which the chosen ratio of approximately one order of magnitude ensures.

The basal interface of the test plate was fully fixed in all spatial directions in all simulations. Surface loads were applied to the truncated cross-section of the spine. All applied loads are physiologically representative; no loads causing joint failure, fracture or separation were considered. The magnitude of the surface load was set to 100 MPa for all load cases; owing to the linear elastic behaviour of the material models and the subsequent normalization of all FEA outputs to the per-simulation maximum, the absolute magnitude has no influence on the results.

Three physiologically relevant load cases were simulated (figure 2C). The inclusion of soft tissue structures in each load case was determined by the direction of force transmission: muscles and ligaments can only transmit tensile forces and were therefore excluded from load cases involving compressive loading of the spine.

*L1 – Axial compression (undeflected):* A compressive surface load was applied to the spine cross-section with the spine in the upright ($0^{\circ }$) configuration. Only the tubercle and spine were modelled. Muscles and ligaments were excluded as they cannot contribute to load transfer under compression.

*L2 – Axial tension (undeflected):* A tensile surface load was applied to the spine cross-section in the upright configuration. All five contact bodies were included in the model: tubercle, spine, inner ligament (referred to as ‘central ligament’ by some studies [12]), outer ligament and muscle ring. The inner and outer ligaments were modelled using the hyperelastic anisotropic model of Gasser *et al.* [36], with material parameters representative of collagenous biological tissues [37], as ligament-specific mechanical data for *Prionocidaris baculosa* are not available in the literature. The muscle ring was modelled using the Ogden hyperelastic model [38] with parameters for smooth muscle from Mo *et al.* [39], adopted as the best available proxy in the absence of sea urchin-specific muscle data. We acknowledge that these soft tissue parameter choices represent approximations and have stated this as a limitation. It should be noted that since the analysis focuses exclusively on the stress response of the calcitic tubercle, the absolute stiffness values of the soft tissue structures are not critical to the conclusions; their role in L2 is solely to transmit the applied tensile force from the spine to the test, thereby loading the tubercle at its ligament and muscle attachment sites.

To validate the simplification of treating soft tissues as isotropic calcite in the SKO and CAIO analyses—which was necessary owing to software constraints on anisotropic optimization—an additional FEA for L2 was performed using the full anisotropic Gasser and Ogden material models for the ligaments and muscle respectively. Stress distributions within the calcitic tubercle were near-identical between the simplified and the anatomically realistic models (see figure 5 and supplementary material, figure S1), confirming that the choice of soft tissue material model does not significantly affect stress patterns in the tubercle, which is the sole subject of the subsequent correlation analysis.

**Figure 3.**
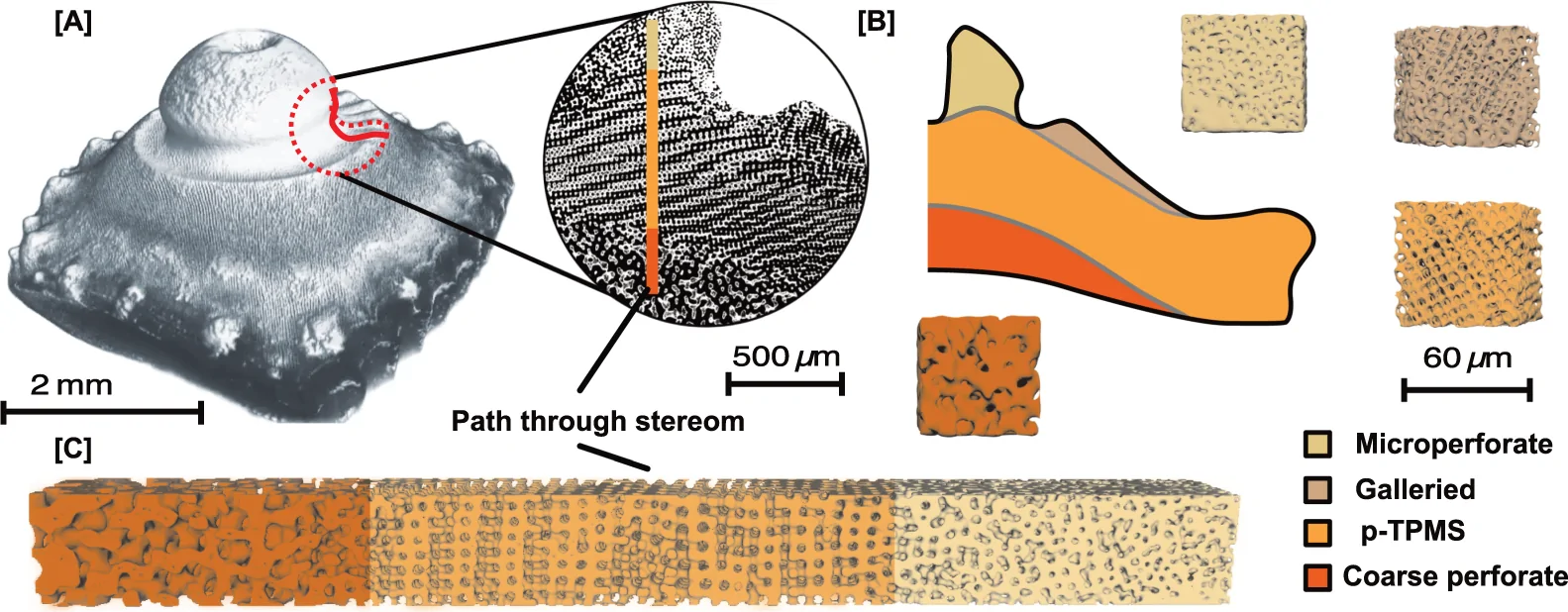
Results of the micro-CT and XRM scans. (A) Overview scan of the tubercle acquired by micro-CT, including a magnified subregion imaged at higher resolution using XRM. The line within the subregion represents a path (path P2) along which data were extracted for subsequent quantitative analysis. The path is colour-coded according to the stereom types it traverses. (B) Qualitative visualization of the stereom distributions within the tubercle as determined from the CT data. Representative stereom cubes extracted from the corresponding regions are shown alongside. (C) Three-dimensional visualization of the complete path shown in (A).

**Figure 4.**
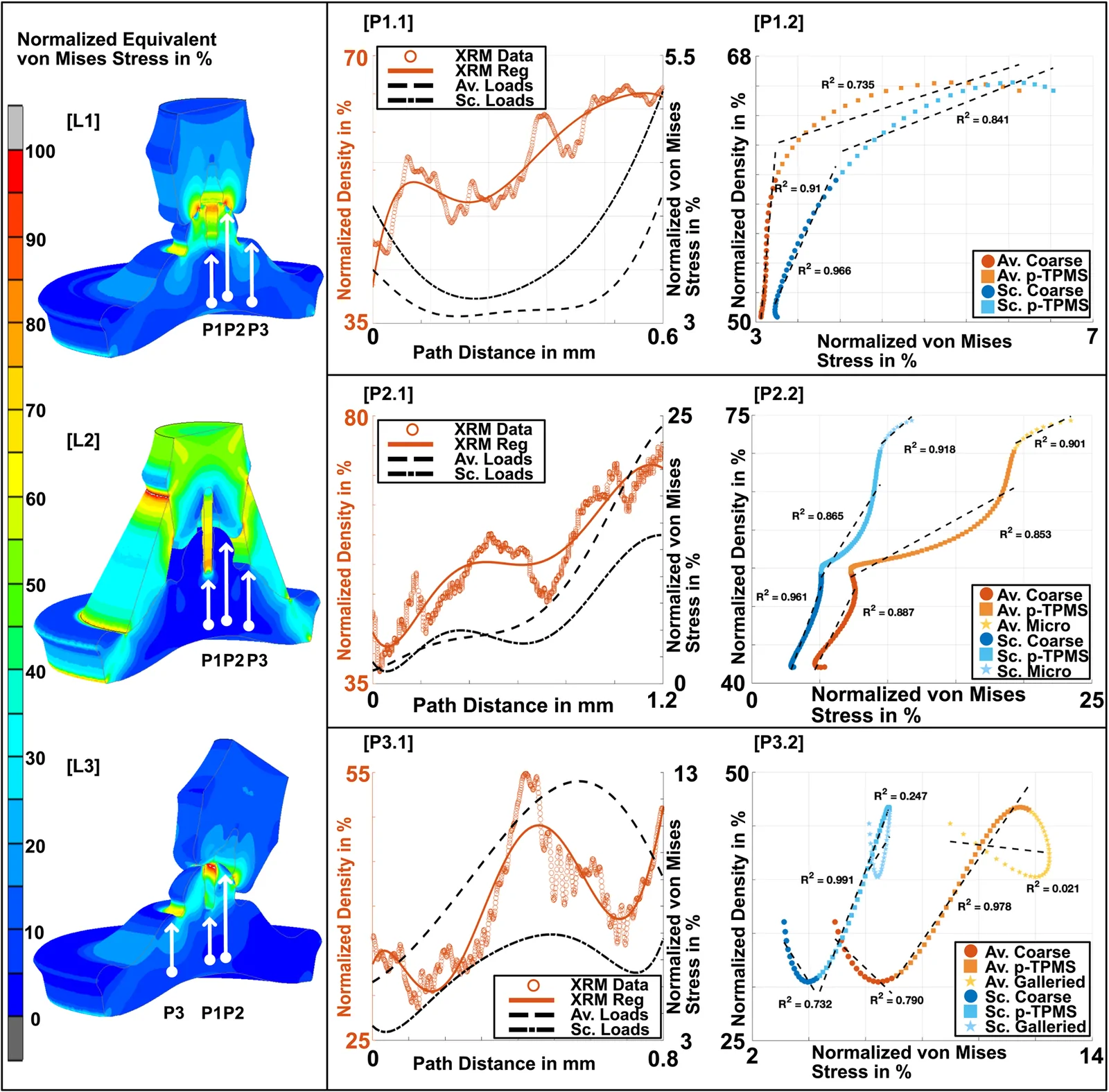
Quantitative correlation between XRM-derived density and FEA-derived von Mises stress. (L1) Normalized von Mises stress distribution from the FEA under compressive loading, showing maximum stresses in the mamelon and elevated bending stresses at the transition to the platform. (L2) Normalized von Mises stress distribution under tensile loading, with highest stresses at the tubercle where the outer and inner ligaments attach, while the mamelon and central regions experience lower stress levels. (L3) Normalized von Mises stress distribution for an axial compressive load applied along the length of the spine in the maximally deflected configuration ($29^{\circ }$ relative to the normal vector of the test surface). The asymmetric stress distribution arises from the geometrical offset between the deflected spine axis and the tubercle's rotational axis, making this the only asymmetric load case; maximum stresses occur in the mamelon and on the platform located behind it from the spine's perspective, with stresses predominantly directed into one wing of the tubercle. In (L1,L2,L3), analysis paths are indicated in white, with dots marking start points and arrowheads indicating direction and end; path P3 is positioned opposite to path P2 in (L3) owing to asymmetric loading. P1.1, P2.1, P3.1: Normalized XRM density values (circles) with fitted sixth-order polynomial regression (orange); the black dashed line indicates the unweighted averaged stress (equation (2.2)), while the dash–dot line shows the weighted stress function (equation (2.3)), yielding an improved fit to the density regression, particularly in the 0.1 to 0.4 interval (P1.1) and the 0.2 to 0.7 range (P3.1). P1.2, P2.2, P3.2: Normalized density plotted against normalized stress (in %) for unscaled (reddish spectrum) and scaled (bluish spectrum) values. Linear regressions for individual path segments are shown as black dashed lines with corresponding $R^{2}$ values, demonstrating stronger linear correlations for the scaled data compared to the unscaled case.

**Figure 5.**
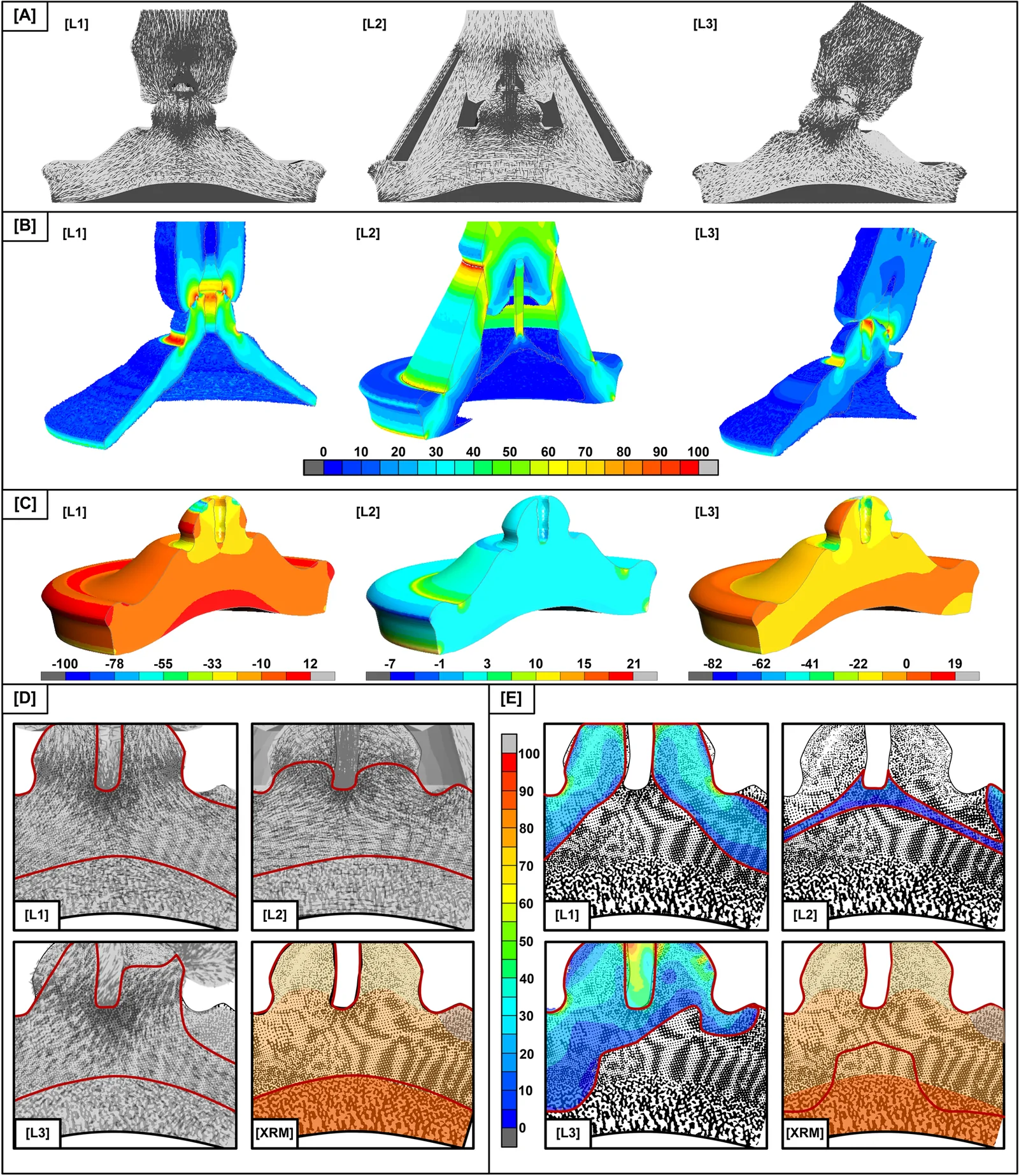
Results of the FEA and qualitative correlation with XRM scans for three load cases (L1 compression, L2 tension, L3 deflected compression). (A) Orientation of the major principal stress in the tubercle–spine systems according to CAIO optimization. Compressive loads are mainly carried by the mamelon and distributed peripherally, whereas tensile loads are accommodated in the apical boss and transferred towards the margins. The basal central region shows no preferred orientation in all three load cases. (B) Von Mises stress distributions in remaining regions after SKO optimization indicate their functional relevance: shanks and mamelon are important for compression, peripheral and central regions for tension. (C) Mean normal stress distributions (normalized to ±100 %) appear also in the basal region across all load cases, showing there predominantly tensile stresses. (D) Overlay of CAIO optimizations and XRM scans (L1 to L3). The basal region lacks preferred orientation and corresponds to coarse labyrinthic stereom. Galleried stereom shows orientation only in L2. The mamelon (microperforate stereom) is associated with compressive loading. Central p-TPMS regions exhibit preferred orientation under all load cases, indicating multi-directional optimization. (E) Structurally relevant regions retained after SKO. Microperforate stereom is mainly adapted to compression, galleried stereom to tension, and p-TPMS to multiple load cases. The coarse labyrinthic stereom is not sensitive towards von Mises stress.

*L3 – Compressive loading of the deflected spine:* A compressive surface load was applied normal to the spine axis in the maximally deflected configuration ($29^{\circ }$ relative to the rotational axis), consistent with the maximum angular displacement observed in the XRM data and with values reported in the literature [12]. Only the tubercle and spine were modelled, for the same reasons as L1. The axial load along the length of the spine leads to an asymmetric load of L3 owing to the deflection of the spine axis relative to the normal vector of the test surface.

In all simulations, the lateral motion of the spine was constrained orthogonally to the loading axis to prevent numerical instabilities arising from the ball-and-socket contact geometry. The von Mises stress, mean normal stress, and major principal stress values were extracted along the three predefined radial paths (P1 to P3; see figure 2) and normalized to each simulation's maximum value.

It should be noted that under the assumption of linear elastic material behaviour, stresses are proportional to the applied load and independent of the absolute value of Young's modulus. Since all FEA stress outputs were normalized to the per-simulation maximum prior to any correlation analysis, the results are dimensionless and insensitive to the specific modulus value chosen. Deformations, which would depend on E, were not analysed in this study.

### 2.6. Optimization analyses

Two iterative optimization methods were applied to identify load-bearing regions and principal stress directions within the tubercle [40]. Both methods are based on the FEA results described in §2.5.

The Soft Kill Option (SKO) method was employed for topology optimization [32]. The conceptual basis of SKO is the Wolff principle [41], which describes adaptive bone remodelling in response to mechanical loading: bone is deposited in regions of high mechanical demand and resorbed in regions of low demand, following the principle that form follows function. While echinoderm stereom does not undergo continuous remodelling through resorption and redeposition in the same manner as vertebrate bone, the Wolff principle is used here strictly as a conceptual analogy for load-adaptive structural organization. This biological observation has been formalized into an established iterative topology optimization method used in structural engineering. In the SKO algorithm, the Young's modulus of each finite element is iteratively modified by comparing the local von Mises stress to a reference stress, $\sigma_{ref}$:

(2.1) $$E_{i+1}=E_{i}+k(\sigma_{i}-\sigma_{ref}),$$

where k is a constant scaling factor and $\sigma_{i}$ denotes the von Mises stress in the respective element. Elements experiencing stresses above the reference threshold are assigned increasing stiffness and retained in the load-bearing structure; elements below the threshold are progressively softened and effectively removed from load transfer, ultimately revealing the principal load paths.

It is important to note that this variation of Young's modulus is a purely algorithmic device and does not represent any physical material change in the stereom. It does not imply changes in porosity, organic matrix content or magnesium incorporation in the calcite. The tubercle is modelled throughout as an isotropic, non-porous continuum, and the resulting stiffness distribution should be interpreted solely as an indicator of mechanically relevant regions under each loading scenario. The approach serves as a hypothesis-testing framework to evaluate whether the natural stereom architecture follows load-adaptive spatial principles analogous to those described for bone remodelling, and to identify which regions of the tubercle are structurally significant under compression, tension and deflection respectively.

The Computer Aided Internal Optimization (CAIO) method was used to investigate internal structural alignment [33]. CAIO iteratively realigns the direction-dependent orthotropic material properties of each finite element with the local major principal stress direction derived from the stress tensor. The resulting optimized element orientations were visualized in ParaView 5.6.2, illustrating the dominant principal stress directions and the inferred load paths within the structure.

### 2.7. Correlation analysis

Quantitative correlations between local stress and density were computed along the three XRM paths P1 to P3. For each path, the normalized XRM-derived density profile $\hat{\rho }(x)$ and the normalized FEA-derived von Mises stress profile $\hat{\sigma }(x)$ were compared using the Pearson correlation coefficient r. Since data points along each path were not always spatially aligned between the FEA and XRM datasets owing to differing mesh resolutions, sixth-order polynomial regressions were fitted to the raw data points of each path prior to correlation, converting them to continuous functions evaluated at identical positions. The polynomial $R^{2}$ values and the Pearson r values reported in table 2 are therefore distinct statistics: the former describes the goodness-of-fit of the polynomial regression to the raw XRM density data points, while the latter describes the correlation between the resulting smoothed density function and the corresponding stress function.

**Table 2.** Pearson correlation coefficients (r) between the normalized XRM-derived density profile and the normalized FEA-derived von Mises stress profile along each of the three radial paths (P1 to P3). Density profiles were obtained from XRM image analysis and fitted by sixth-order polynomial regression; stress profiles were extracted from the FEA along the same paths and normalized to the per-simulation maximum. Two stress measures are compared: the unweighted arithmetic mean across load cases L1 to L3 (Av., equation (2.2)), and a weighted combination (Sc., equation (2.3)) in which the contribution of each load case is scaled by empirically determined weights a (L1), b (L2), and c (L3), listed in the lower portion of the table. The weights were determined independently for each path by maximizing the Pearson correlation between the weighted stress profile, reflecting the possibility that different structural regions are preferentially adapted to specific loading scenarios. The scaling parameters used for each path are listed in the lower portion of the table. Weighted correlations consistently exceed unweighted correlations across all paths, with path 2 yielding the highest overall coefficients, attributable to its greater length and the higher proportion of p-TPMS stereom traversed.

	PC path 1	PC path 2	PC path 3
Av. L1–L3	0.58	0.94	0.76
Sc. L1–L3	0.75	0.95	0.84
	**SC path 1**	**SC path 2**	**SC path 3**
a	1.5	1.0	1.0
b	2.0	2.0	7.0
c	3.0	0.1	1.0

Two stress functions were compared against the density profile for each path. The first is the unweighted arithmetic mean across all three load cases (equation (2.2)); the second is a weighted combination (equation (2.3)):

(2.2)LAv=L1+L2+L33,(2.3)LSc=a⋅L1+b⋅L2+c⋅L3a+b+c,

where a, b and c are empirically determined scaling factors for load cases L1, L2 and L3, respectively, listed in table 2. Weights were determined independently for each path by maximizing r, reflecting the possibility that different structural regions are preferentially adapted to specific loading scenarios. Linear regression analyses were additionally performed within stereom-defined subregions (coarse labyrinthic (irregular perforate), p-TPMS, microperforate, galleried) along each path, yielding local slopes and $R^{2}$ values that characterize the linearity of the density–stress relationship within each stereom type independently. Weighted multi-load correlations achieved a mean Pearson coefficient of r=0.92±0.04 across all paths.

## 3. Results

### 3.1. X-ray microscopy

High-resolution XRM scans revealed a complex internal ultrastructure of the tubercle in *Prionocidaris baculosa* (figure 3). Four basic stereom types were distinguished based on pore morphology and spatial arrangement following [1]: coarse labyrinthic (or irregular perforate), rectilinear (mostly represented by p-TPMS), microperforate and galleried stereom. The coarse labyrinthic stereom occurred in the basal regions of the test plate, the p-TPMS type dominated the central boss and plate peripheries, microperforate structures formed the mamelon, and galleried stereom aligned with the apical ligament attachment zones [1]. The size and orientation of the pores varied systematically along the apical–basal axis, indicating a functionally graded architecture.

### 3.2. Finite-element analysis

Finite-element simulations under compression, tension, and deflection showed distinct stress distributions corresponding to functional regions (figure 4). Under compression (L1), the highest von Mises stresses occurred within the apical mamelon (normalized 100%), decreasing towards the basal coarse-perforate (labyrinthic) region (< 40%). Tensile loading (L2) resulted in elevated stresses at ligament attachment sites on the boss (≈ 40–60%), whereas the mamelon exhibited markedly lower stress magnitudes (< 30%). In the deflected load case (L3), an axial compressive load was applied along the length of the spine, which was oriented at $29^{\circ }$ relative to the normal vector of the test surface. The resulting stress distribution within the tubercle is asymmetric, as the geometrical offset between the deflected spine axis and the tubercle's rotational axis directs stresses preferentially into one wing of the tubercle.

### 3.3. Orientation and topology optimization (CAIO and SKO)

CAIO analysis revealed a consistent collective alignment of elements along principal stress directions (figure 5A). In all load cases, the strongest orientational coherence occurred in the mamelon and mid-boss regions. In the apically located galleried stereom regions, load transfer is predominantly unidirectional, which becomes evident under tensile loading. The basal irregular-perforate (labyrinthic) regions showed little alignment, reflecting their probably secondary structural function. The degree of orientational coherence was the highest under compression and deflection, aligning with observed p-TPMS trabecular orientations.

The SKO optimization revealed key load-bearing regions identified in the FEA (figure 5B). Material removal occurred predominantly in the basal and apical areas, leaving a continuous load path from the mamelon through the platform to the boss. This pattern closely matched the p-TPMS and galleried regions observed in XRM cross-sections.

### 3.4. Quantitative correlation between density and stress

To quantify the relationship between stereom organization and local mechanical demand, XRM-derived density profiles were compared with FEA-derived von Mises stress distributions along the three radial paths P1–P3 (figure 4; path locations shown in figure 3). Two distinct statistical measures are reported. First, sixth-order polynomial regressions were fitted independently to the raw XRM density data points and to the FEA stress data points along each path, yielding goodness-of-fit values of $R^{2}=0.86$ to 0.90 for the density profiles. This fitting step was necessary to align the two datasets spatially, as the FEA mesh and XRM image sampling positions do not coincide. Second, the Pearson correlation coefficient r between the resulting smoothed density polynomial $\hat{\rho }(x)$ and the smoothed stress polynomial $\hat{\sigma }(x)$ was computed along each path, yielding r=0.58 to 0.94 for the unweighted mean stress (equation (2.2)) and r=0.75 to 0.95 for the weighted stress combination (equation (2.3)); table 2. The $R^{2}$ values therefore characterize the quality of the density profile fit, while the Pearson r values characterize the strength of the density–stress correlation; these are independent quantities. Applying weighted combinations of load cases according to equation (2.3) further improved the correlations for all paths, with the scaling parameters listed in table 2.

Linear regression analyses within stereom-defined subregions supported these findings (figure 4, panels P1.2, P2.2, P3.2). Coarse labyrinthic regions exhibited moderate linearity, with $R^{2}$ values ranging from 0.54 to 0.91 and slopes of approximately 3 to 5. The strongest proportionality was observed in p-TPMS regions, which showed $R^{2}$ values between 0.89 and 0.99 and slopes of approximately 6 to 7. Microperforate regions also displayed positive correlations, although with weaker trends ($R^{2}=0.73$ to 0.90). By contrast, the galleried stereom exhibited low linearity ($R^{2}<0.25$), indicating a tendency towards stress dissipation rather than stress concentration.

Across all load cases, regions of higher local density consistently coincided with elevated von Mises stresses and major principal stress trajectories, supporting the hypothesis that stereom organization reflects mechanical adaptation. The weighted multi-load correlations yielded an overall mean Pearson coefficient of r=0.92±0.04.

### 3.5. Qualitative comparison of X-ray microscopy and simulation outputs

The overlay of XRM images with CAIO and SKO optimization maps confirmed spatial agreement between structural density and functional load areas (figure 5). The p-TPMS zone shows a clear orientation of the major principal stress (CAIO) and higher von Mises stress values (SKO) for all load cases. The CAIO alignments parallelled the orientations of trabecular networks in these regions, including within the microperforate mamelon, which is subjected to high pressure values.

Tension loading, according to L2, leads to a major principal stress orientation in the galleried stereom zone. In addition, the galleried zones match closely with the load-bearing areas preserved in the SKO simulation.

In the basal coarse-irregular-perforated (labyrinthic) zone, the principal stress analysis does not show any orientation. Low von Mises stress values lead to the elimination of this area during SKO optimization, which coincides with higher mean normal stress values. Together, these analyses indicate that the echinoid tubercle represents a hierarchically organized, functionally graded architecture optimized for multi-axial loading.

## 4. Discussion

This study provides new insights into the hierarchical organization and mechanical behaviour of the echinoid tubercle by explicitly linking stereom microarchitecture to local stress distributions within a load-bearing joint. By combining high-resolution XRM, FEA, and computational optimization approaches (CAIO and SKO), we demonstrate that the interambulacral plate having tubercle of *Prionocidaris baculosa* comprises a spatially organized assemblage of distinct stereom types whose distribution corresponds closely to the mechanical demands imposed by compression, tension and deflection.

XRM analyses revealed four principal stereom types—coarse labyrinthic (irregular perforate), p-TPMS, microperforate and galleried—occupying well-defined regions of the plate. This organization matches the stereom distributions described for some other echinoids and cidaroids and supports the long-standing interpretation that stereom types are functionally differentiated rather than randomly distributed [1,11,13,16]. Despite minor differences, the recurrence of broadly similar stereom organizational patterns across echinoid clades [1] suggests similar structural solutions shaped by long-term evolutionary pressures towards mechanical optimization.

The coarse disordered labyrinthic (irregular perforate) stereom at the base of the plate probably acts as a structurally compliant boundary separating the rigid test from the underlying coelomic cavity, while the p-TPMS-like stereom in the mid-tubercle and in the plate periphery region provides relatively isotropic stiffness suitable for multi-directional load transfer. Microperforate stereom in the mamelon coincides with regions of elevated compressive stress, whereas galleried stereom aligns with ligament attachment sites and principal tensile directions, consistent with earlier functional interpretations [1]. Together, these findings highlight a coordinated spatial arrangement that enables both mechanical robustness and controlled flexibility within the joint.

Quantitative comparison of XRM-derived density profiles with FEA-derived stress distributions revealed strong correlations along all analysed paths, with Pearson correlation coefficients reaching up to 0.95 when multiple load cases were scaled. Linear regression within stereom-defined subregions further showed that p-TPMS-like stereom exhibits the strongest proportionality between density and stress, whereas galleried stereom displays low linearity, consistent with a role in stress dissipation rather than stress concentration [4]. Furthermore, the second path exhibited the highest correlations. This may be attributed to its greater length, the higher proportion of TMPS, and the smaller contribution of marginal regions. According to Raman *et al.*, the density of the stereom decreases towards the margins, presumably owing to muscle attachments or other tissues interwoven with the stereom [18]. Although the paths were therefore deliberately positioned away from the margins, these regions may still influence the overall correlation along the path. These results indicate that stereom differentiation reflects functional integration of multiple load-bearing strategies within a single ossicle rather than optimization for a single dominant load case.

The results of computational optimization provide additional support for this interpretation. Both SKO and CAIO simulations reproduced key aspects of the natural architecture, with optimized material distributions and alignment patterns converging on regions occupied by p-TPMS-like and microperforate stereom. Similar correspondences between computationally predicted stress trajectories and echinoderm skeletal organization have been reported previously for echinoid spines and test plates [21,26]. While biological systems are not expected to achieve ‘mathematical’ optima, the partial convergence between simulated and observed structures suggests that echinoid skeletons approximate mechanically efficient configurations shaped by incremental evolutionary adaptation rather than direct optimization.

A limitation of the present study is that the quantitative analyses are based on a single tubercle–spine assembly, selected from a set of specimens screened by micro-CT on the basis of structural completeness and geometric symmetry. While cidaroid stereom microarchitecture is known to be conserved across individuals and taxa [1,11,16], future studies incorporating multiple specimens and species would allow direct quantification of inter-individual variability and further test the generality of the structure–stress correlations reported here. Similarly, the maximum deflection angle of $29^{\circ }$ was determined geometrically from the CAD reconstruction of the specific interambulacral tubercle analysed and is consistent with the morphological range reported by Smith *et al.* [12]; direct kinematic measurements of spine deflection in living cidaroids are not available in the published literature, and future studies combining *in vivo* imaging with structural analysis would strengthen the physiological grounding of the load cases applied here.

Recent work on *Cidaris rugosa* has emphasized that echinoid ossicles function as composite materials composed of multiple coexisting microarchitectures, including disordered and ordered p-TPMS domains, each contributing distinct mechanical properties [11]. Our results for *P. baculosa* are fully consistent with this view and extend it by explicitly linking stereom heterogeneity to local stress fields within a functional joint. Together, these studies suggest that internal architectural heterogeneity is a conserved and functionally relevant feature of cidaroid skeletons, enabling local specialization while maintaining overall structural integrity.

Importantly, mechanical adaptation in echinoderm skeletons operates within developmental and physiological constraints. Stereom porosity must accommodate cellular activity, mutable collagenous tissues and metabolic exchange, implying that its architecture reflects a balance between mechanical performance and biological function rather than purely mechanical optimization [1,16]. The observed correspondence between stress distributions and stereom organization therefore reflects a multi-functional compromise shaped by both physical and biological requirements.

Beyond their relevance for understanding echinoderm skeletal organization, the present findings also have implications for the abstraction of biological design principles into engineering contexts. Importantly, the tubercle of *Prionocidaris baculosa* does not represent a single optimized structure, but rather a spatially integrated assembly of distinct microarchitectures that collectively accommodate multiple, partly competing mechanical requirements. This principle of functional differentiation within a continuous material framework contrasts with many engineered lightweight structures, which typically rely on uniform lattice geometries or discrete material interfaces [42,43].

The observed correlation between local stress states and stereom type suggests that mechanical performance is achieved not through global maximization of stiffness or strength, but through regional tuning of porosity, connectivity and orientation. In particular, the combination of highly isotropic, p-TPMS-like architectures in regions exposed to multi-directional loading, denser microperforate stereom in compression-dominated zones, and galleried structures aligned with tensile load paths highlights a strategy of embedding multiple mechanical functions within a single material system. From a biomimetic perspective, this supports the concept of functionally graded cellular materials in which geometry, rather than material composition, governs mechanical behaviour.

Recent advances in additive manufacturing make such concepts increasingly accessible. Additive processes allow the fabrication of components with locally varying lattice architectures, pore sizes and strut orientations, even when using a single base material. The stereom organization observed here provides a biologically grounded reference for how such gradients might be arranged in response to anticipated load cases, particularly in joint-like structures where compression, tension, and bending occur simultaneously. Potential application areas include lightweight mechanical joints, prosthetic interfaces and architected materials for load-bearing components where damage tolerance and stress redistribution are critical.

At the same time, the echinoid stereom underscores that biological load-adaptive architectures are shaped by constraints beyond mechanics alone. The necessity to maintain permeability for cellular processes, growth and repair imposes limits on densification and alignment that are absent in purely technical systems. Translating stereom-inspired concepts into engineering applications therefore requires careful abstraction of underlying principles—such as hierarchical organization, regional specialization and redundancy—rather than direct geometric replication.

In this sense, the echinoid plate serves less as a template for specific designs and more as a model system that illustrates how complex mechanical functions can emerge from the integration of multiple microarchitectural motifs within a single mineralized structure. Incorporating such principles into engineering design may enable the development of lightweight, mechanically resilient components that balance strength, robustness and adaptability in ways that are difficult to achieve using homogeneous architectures alone.

## Supplementary Material



## Data Availability

The dataset required to replicatethe study is available from [44]. Supplementary material is available online [45].
