# Supplementary material for: Stress-correlated stereom organization in the echinoid tubercle reveals hierarchical mechanical integration
Source: J R Soc Interface. 2026 Oct 14;23(243):20260592. doi: 10.1098/rsif.2026.0592 (PMC13682557; doi:10.1098/rsif.2026.0592)
Supplement: Supplementary file 1 [file rsif.2026.0592.zip › rsif.2026.0592/rsif20260592_si_001.pdf]

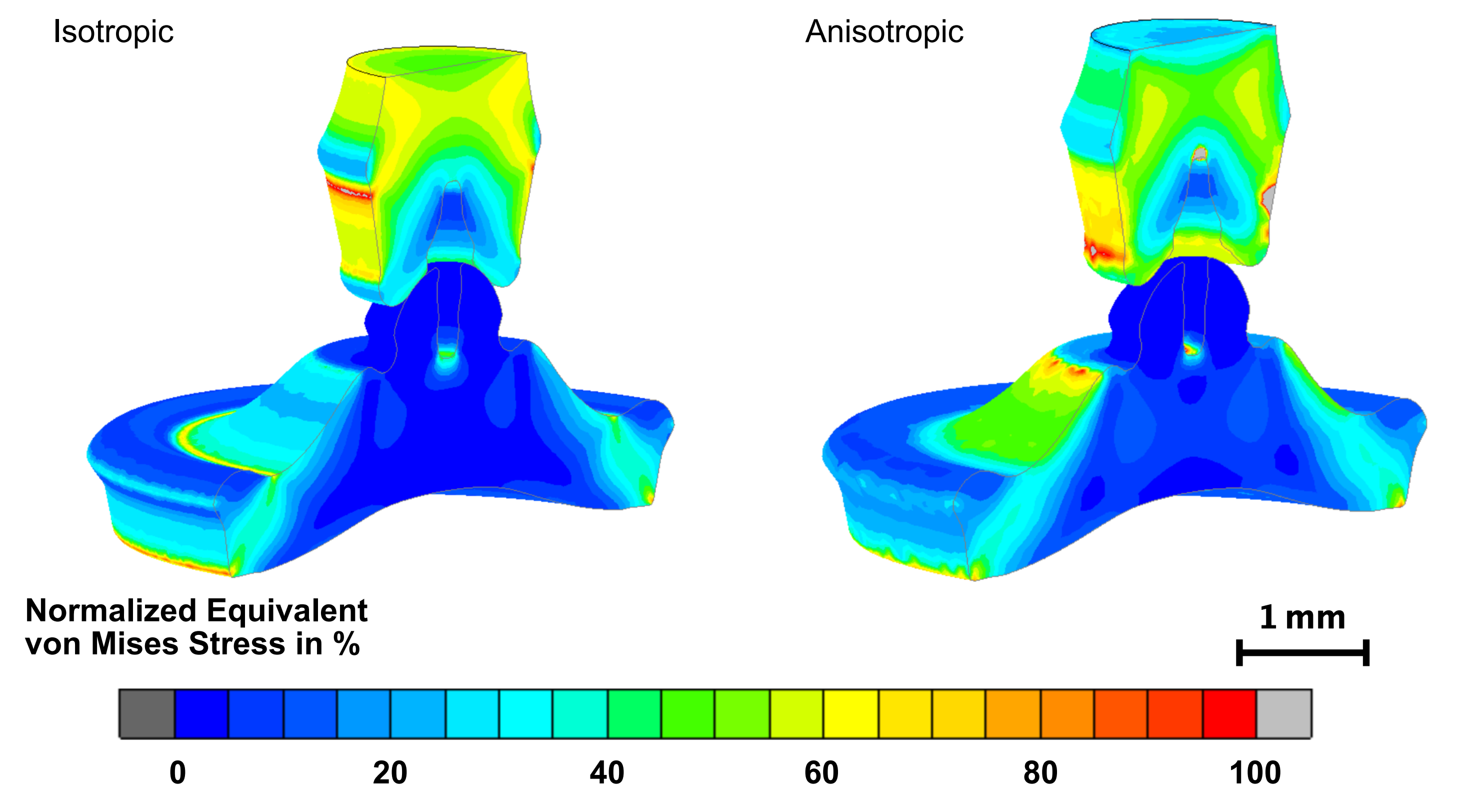
Isotropic
Anisotropic
Normalized Equivalent
von Mises Stress in %
1 mm
0
20
40
60
80
100
